# Current pathophysiological concepts in cerebral small vessel disease

**DOI:** 10.3389/fnagi.2014.00024

**Published:** 2014-03-24

**Authors:** Fred Rincon, Clinton B. Wright

**Affiliations:** ^1^Department of Neurology, Thomas Jefferson UniversityPhiladelphia, PA, USA; ^2^Department of Neurosurgery, Thomas Jefferson UniversityPhiladelphia, PA, USA; ^3^Evelyn F. McKnight Brain Institute, Department of Neurology, University of MiamiMiami, FL, USA; ^4^Department of Epidemiology and Public Health, University of MiamiMiami, FL, USA; ^5^Neuroscience Program, University of MiamiMiami, FL, USA

**Keywords:** stroke, leukoaraiosis, silent brain infarction, vascular cognitive impairment, Alzheimer’s disease

## Abstract

The association between cerebral small vessel disease (SVD) – in the form of white matter lesions, infarctions, and hemorrhages – with vascular cognitive impairment (VCI), has mostly been deduced from observational studies. Pathological conditions affecting the small vessels of the brain and leading to SVD have suggested plausible molecular mechanisms involved in vascular damage and their impact on brain function. However, much still needs to be clarified in understanding the pathophysiology of VCI, the role of neurodegenerative processes such as Alzheimer’s disease, and the impact of aging itself. In addition, both genetic predispositions and environmental exposures may potentiate the development of SVD and interact with normal aging to impact cognitive function and require further study. Advances in technology, in the analysis of genetic and epigenetic data, neuroimaging such as magnetic resonance imaging, and new biomarkers will help to clarify the complex factors leading to SVD and the expression of VCI.

## INTRODUCTION

Stroke remains the No. 1 cause of disability and the fourth leading cause of death in the US (Roger et al., 2012). Prevention strategies aimed at treating modifiable risk factors have been advocated by clinicians and epidemiologists (Rincon and Sacco, 2008). Among the important causes of stroke, hypertension-related small vessel disease (SVD) and cerebral amyloid angiopathy (CAA) are the most common forms, and have generated significant academic interest because of their sinister impact on brain function. Understanding the pathophysiological mechanisms involved in SVD and possible treatments has remained elusive (Pantoni, 2010). In this review, we sought to summarize recent advances in the understanding of the pathophysiological mechanisms of SVD.

## SMALL VESSEL DISEASE

The term SVD refers to the syndrome of clinical, cognitive, neuroimaging, and neuropathological findings thought to arise from damage to (a) small arteries, (b) arterioles, (c) capillaries, and (d) small veins and venules in the brain (Moody et al., 1995). SVD preferentially affects the vessels of the basal ganglia, peripheral white matter, leptomeningeal arteries, thalamic and cerebellar white matter vessels, and vessels of the brainstem. Cortical vessels are usually not involved in SVD (Thal et al., 2003).

Small vessel disease is an important clinico-pathological condition as it is the cause of 20% of strokes worldwide, and the most common cause of vascular and mixed dementia [vascular dementia (VaD) and Alzheimer’s disease (AD); Pantoni, 2010; Gorelick et al., 2011]. Dementia is currently a pressing public health problem as numbers of affected patients increase steadily. Vascular brain injury is the second most common cause of dementia after AD and a defining feature of vascular cognitive impairment (VCI; Rincon and Wright, 2013). AD commonly coexists with cerebrovascular disease in the elderly population. Though the risk factors for both SVD and AD overlap (Dichgans and Zietemann, 2012), the differentiation on clinical grounds is often difficult (Schneider et al., 2007).

Recently, studies have emphasized on the comorbidities associated with AD and VaD. Established risk factors for both VaD and AD are age, smoking, physical inactivity, obesity, diabetes mellitus, stroke, and peripheral arterial disease (Dichgans and Zietemann, 2012). Brains from AD patients exhibit more cerebrovascular lesions than non-AD patients (Jellinger and Attems, 2005). Pathological examination of brains from AD patients reveal higher prevalence of lacunes, white matter lesions (WMLs), microbleeds, and CAA (Jellinger and Attems, 2005). Pathological changes seen in AD have led authors to believe that vascular brain damage is an important component of AD pathophysiology (de la Torre, 2002). Almost all brains of AD patients have CAA (Jellinger, 2002). This suggests a common β-amyloid-based pathogenesis for the disease. However, despite this molecular relationship, CAA is a different entity from AD as less than 50% of CAA cases meet the pathologic criteria for AD and >75% of patients with AD have mild or no CAA at all (Vinters, 1987; Ellis et al., 1996).

Cerebral amyloid angiopathy-related impairments in cerebral perfusion may be responsible for subcortical WMLs and microscopic damage seen in the disease (Gurol et al., 2006; Holland et al., 2008; Viswanathan et al., 2008). Some studies have suggested that advanced CAA is associated with a larger burden of WMLs as compared to healthy controls or AD patients (Gurol et al., 2006; Holland et al., 2008). Interestingly, CAA induced cognitive impairment may be a reflection of WMLs independent of brain hemorrhages (Viswanathan et al., 2008). It appears that CAA-related WMLs spread through similar areas affected by hypertensive SVD. However, there is some suggestion that CAA induced WMLs may be preferentially seen in the posterior white matter (Zhu et al., 2012). In addition, pathologic studies have suggested that cortical microinfarcts are common in CAA (Soontornniyomkij et al., 2010). The presence of these microinfarcts may be unrelated to classic vascular risk factors. The pathophysiological mechanisms thought to be involved are impaired autoregulation, smooth muscle damage, and capillary occlusion (Shin et al., 2007; Smith et al., 2008; Thal et al., 2009).

Although pathological studies have shown a significantly higher prevalence of vascular pathology in AD patients (Jellinger and Attems, 2005) and despite stroke being a frequent occurrence in elderly AD patients (Honig et al., 2003), the pathophysiological mechanisms and impact of these cerebrovascular abnormalities on cognitive decline in AD remains unclear.

### DIAGNOSIS

Because small vessel damage cannot be readily visualized *in vivo*, the effect of SVD on the brain parenchyma is usually inferred from findings on computed tomography (CT) or magnetic resonance imaging (MRI), and these changes are considered the hallmarks of the disease. As such, SVD is often equated with brain parenchymal lesions. However, it may be beneficial to broaden the definition of this phenotype of SVD to include vascular damage prior to ischemic injury, and use other measures of vascular dysfunction – such as measures of cerebral autoregulation. To date such studies have been limited (Pantoni, 2010). This broader view of SVD allows consideration of plausible therapeutic interventions aimed at modulating the progression of disease before irreversible damage is done.

Consequences of SVD in the brain parenchyma include lesions located in the subcortical structures such as lacunar infarcts, WMLs, and deep hemorrhages (large sub-cortical hemorrhages and microbleeds; Pantoni, 2010; **Table 1**). The prevalence of silent brain infarcts has varied across study populations, across imaging techniques, and the definition of infarct used (Vermeer et al., 2007). Several large-population-based studies have reported prevalence estimates of 8–28%, with differences mainly explained by age (Vermeer et al., 2007). Microscopic damage, which escapes the resolution of most CT and MRI machines, is even more prevalent (Launer et al., 2011). Microscopic brain infarcts (MBI) have been seen in up to 68% of community-based participants (Launer et al., 2011). The therapeutic implications of SVD are important, as SVD is the cause of up to 25% of ischemic strokes and the majority of intracerebral hemorrhages (Petty et al., 2000). No specific therapy exists in the acute setting for strokes caused by SVD. Perhaps more importantly, there is no suggestion that cornerstone treatments of ischemic stroke such as aspirin, thrombolysis, or admission to a stroke unit or neuroscience center are associated with better outcomes in this subset of patients than for other stroke subtypes (Cocho et al., 2006; Pantoni, 2010). Beyond control of blood pressure and statin treatment (Dufouil et al., 2005; Mok et al., 2009), there appears to be no effective therapy to limit the extent and progression of SVD-related stroke. Thus, an opportunity exists to design novel prevention strategies to treat SVD if the pathophysiological factors that distinguish it from large vessel disease and cardiac causes of stroke can be identified. Indeed, chronic hypoperfusion, ischemia, and, finally, necrosis of brain tissue are often associated with SVD and include a number of types of injuries that serve as markers of disease severity, and relate to the risk of poor outcomes and prognosis in a number of clinical settings (Wright et al., 2008).

**Table 1:** Glossary of definitions of SVD phenotypes.

Phenotype	Known genetic mechanisms
***Deep brain infarcts***
**Acute.** Small sub-cortical infarcts 3–20 mm in diameter seen in CT or MRI. These are best defined in the DWI sequence and appear hyper-intense. Usually located in the territory of one perforating arteriole. Imaging and clinical features suggest the event occurred in the immediate previous weeks.	Novel risk-associated SNP *rs2208454* on chromosome 20p12 (Debette etal., 2010). This SNP is located in intron 3 of MACRO domain containing 2 (MACROD2) gene and in the downstream region of fibronectin leucine-rich transmembrane protein-3 (FLRT3) gene (Debette etal., 2010). These regions have been implicated in the regulation of growth factor signaling, angiogenesis, and neurogenesis, and ae associated with decreased risk of cerebral infarction seen on MRI
**Chronic (lacunes).** A round or ovoid sub-cortical fluid filled cavity of 3–20 mm in diameter seen in CT or MRI. These lesions are beds defined in the FLAIR sequence and appear hypointense. Occasionally they have a hyperintense surrounding rim. These lesions are consistent with previous acute small sub-cortical infarcts or hemorrhages in the territory of one perforating arteriole.
***White matter lesions***
Rounded areas of decreased attenuation on CT, increased signal on T2-weighted and FLAIR in the periventricular and white matter of the cerebral hemispheres, basal ganglia (deep gray matter), pons, and brainstem and cerebellum.	Six novel SNPs were identified on one locus of chromosome 17q25 (Fornage etal., 2011). These novel SNPs encompassed six known genes including the WW domain binding protein gene (WBP2), two tripartite motif-containing genes (TRIM65 and TRIM47), the mitochondrial ribosomal protein L38 gene (MRPL38), the Fas-binding factor 1 gene (FB1), and the acyl-coenzyme A oxidase 1 gene (ACOX1; Fornage etal., 2011). The genes are known for being involved in a broad range of biological processes including innate immunity, cell cycle regulation, vesicular trafficking, neuroprotection, and apoptosis (Vandeputte etal., 2001; Meroni and Diez-Roux, 2005; Ozato etal., 2008).
***Cerebral microbleeds***
Small punctuate areas up to 10 mm in diameter of hypointensity in T2*, gradient echo, or susceptibility-weighted imaging. These lesions correspond to small collections of hemosiderin-laden macrophages around small perforating vessels.******	A pattern of deep sub-cortical microbleeds has been associated with vascular risk factors, and a greater burden of WMLs, deep brain infarcts, and a lobar pattern associated with CAA and the ApoE4 genotype (Vernooij etal., 2008).

*CT, computed tomography; MRI, magnetic resonance imaging; DWI, diffusion weighted imaging; FLAIR, fluid attenuated inversion recovery; SNP, single nucleotide polymorphism; WML, white matter lesions.*

For the purpose of this review, we will concentrate on SVD caused by traditional vascular risk factors (sporadic SVD), rather than those caused by genetic abnormalities or associated with systemic conditions [i.e., cerebral autosomal dominant arteriopathy with sub-cortical infarcts and leukoencephalopathy (CADASIL), cerebral autosomal recessive arteriopathy with sub-cortical infarcts and leukoencephalopathy (CARASIL), collagen 4 AI gene (COL4AI) mutations, Fabry’s, hereditary endotheliopathy with retinopathy, nephropathy, and stroke (HERNS), and small vessel arteritis].

### DEEP BRAIN INFARCTS

This term refers to small sub-cortical infarcts of 3–20 mm in diameter identified on either CT or MRI (Norrving, 2003; Wardlaw et al., 2013). Deep brain infarcts, often referred to as lacunar strokes in the clinical setting, account for 20–30% of all stroke sub-types, and have an incidence of about 33 per 100,000 persons/years (Sudlow and Warlow, 1997). Acutely, deep brain infarcts are better detected by MRI than by CT, and appear hyperintense on diffusion-weighted imaging (DWI), and within hours to days on T2-weighted imaging or fluid attenuated inversion recovery (FLAIR) sequences (Patel and Markus, 2011). Chronic deep brain infarcts appear hypo-intense on T1 and FLAIR, and often have a hyper-intense rim around them on the latter sequence (Patel and Markus, 2011). Once macrophages have removed infarcted tissue, irregular cavities are left with surrounding gliosis, and lipid-rich and hemosiderin-rich macrophages are left in surrounding gliotic tissue along with extravasated plasma proteins, fibrinoid necrosis, and vascular fragments (Fisher, 1968). Many risk factors associated with deep brain infarcts, such as older age, and particularly hypertension, but also diabetes mellitus, smoking, excess alcohol consumption, and dyslipidemia, are shared with those of superficial infarcts. Some epidemiological studies suggest these risk factors may not be as important as in infarcts related to atherosclerotic arteriopathy (Jackson and Sudlow, 2005; Jackson et al., 2010).

While CADASIL, CARASIL, Fabry’s disease, and a number of other genetic forms of SVD are known, the genetic underpinning of most SVD is poorly understood. However, recent studies are beginning to show that genetic factors likely play a role. A genetic mechanism leading to SVD has been suggested as underlying some deep brain infarcts (Jackson et al., 2010). For example, a recent population-based genome-wide association study (GWAS) on covert MRI-defined brain infarcts found the novel risk-associated single nucleotide polymorphism (SNP) *rs2208454* on chromosome 20p12 (Debette et al., 2010). This SNP is located in intron 3 of MACRO domain containing 2 (MACROD2) gene and in the downstream region of fibronectin leucine-rich transmembrane protein-3 (FLRT3) gene (Debette et al., 2010). These regions have been implicated in the regulation of growth factor signaling, angiogenesis, and neurogenesis, and are associated with decreased risk of cerebral infarction seen on MRI (Debette et al., 2010).

Deep brain infarction is associated with classical clinical syndromes (also named lacunar syndromes; Donnan and Norrving, 2009) and are closely associated with radiological evidence of ischemia, although some authors have demonstrated that the clinical syndrome may not be entirely predictive of the lesion or location (Gan et al., 1997; Arboix et al., 2010). The presence of sub-clinical evidence of brain ischemia, or “silent” brain infarcts (Vermeer et al., 2007), makes it difficult to identify associated risk factors, underlying mechanisms, and potential therapies for intervention. Though deep brain infarcts have an overall better prognosis (Norrving, 2003), they have a higher rate of recurrence and affected individuals are at greater risk of developing cognitive impairment, depression, and long-term functional decline (Miyao et al., 1992; Samuelsson et al., 1996; Yamamoto et al., 2002; Vermeer et al., 2003, 2007; Baezner et al., 2008).

Recently, there has been a growing interest in the clinical significance of deep brain infarcts and WMLs as causes of VCI. It is important to note, that the classic lacunar syndromes did not include cognitive impairment as a feature or dedicated syndrome (Norrving, 2003). However, small deep brain infarcts are known to cause so-called “strategic infarct dementia” (Tatemichi et al., 1992) and the lesion burden has also been associated with dementia risk (Koga et al., 2009). In one study, the presence of thalamic lacunes was associated with poor global cognitive performance, low motor activity and executive function performance; and the presence of lacunes in the pallidum or putamen was associated with memory dysfunction (Benisty et al., 2009). Deep brain infarcts have been associated with a number of outcomes that are relevant to VCI, including cognitive decline, dementia, gait disturbance, urinary incontinence, and disability (Miyao et al., 1992; Samuelsson et al., 1996; Yamamoto et al., 2002; Vermeer et al., 2003, 2007; Baezner et al., 2008), making the term “silent infarct” an inappropriate and misleading term, given these poor outcomes (Pantoni, 2003; Hachinski, 2008).

### WHITE MATTER LESIONS

This phenotype of SVD represents a different entity than deep brain infarcts, however, they often coexist (Pantoni, 2010).

The prevalence of WMLs in the white population is 80% or greater in those 60 years old or older (de Leeuw et al., 2001), and seen more in women as compared to men (de Leeuw et al., 2001). Before the advent of MRI, WMLs were seen on CT imaging as x-ray attenuation in white matter areas and described in the literature by Hachinski et al. (1987) as “leukoaraiosis.” On MRI, WMLs are seen as white matter hyperintensities (WMH) on T2 and FLAIR sequences (Wardlaw et al., 2013). Such WMLs are seen in white matter tracts surrounding the ventricular system, though they are also seen in other areas and in the immediate subcortical white matter. Magnetic resonance-based diffusion tensor imaging (DTI) provides a measure the diffusion of water in white matter tracts, allowing researchers to examine the patency of axonal pathways in patients with SVD (de Laat et al., 2011). Studies using DTI have shown that white matter integrity is compromised immediately outside WMH lesions (Maillard et al., 2011), suggesting that visible lesions are indicative of wider injury. Additional promising MRI-based techniques for the study of WMLs in SVD include magnetization transfer (MTI) and high-field-MRI (Fazekas et al., 2005; Bastin et al., 2009; Kang et al., 2010), as the severity of tissue changes associated with incidental WMLs in these patients cannot be sufficiently determined by conventional MRI.

Population-based studies have demonstrated a strong association between both age and hypertension and WMLs (Enzinger et al., 2007). Similarly, pathological studies have shed some light on the association between ischemia and WMLs as well (Enzinger et al., 2007). Common pathological findings in WMLs are: mild perivascular alterations to large areas with variable loss of fibers, multiple small cavitations, and marked arteriolosclerosis (Fazekas et al., 1993). In addition, WMLs have a variety of pathological correlates depending on the severity of ischemic tissue damage: myelin pallor, gliosis, axonal loss, complete nerve fiber destruction (Fazekas et al., 1993), and, in the worst cases, blood–brain barrier disruption and loss of endothelium (Young et al., 2008). The tissue surrounding WMLs may be highly “active” with foam cells, activated astrocytes, and microglia (Fazekas et al., 1993). Up-regulation of inflammatory markers seen in these areas, including apolipoprotein E (ApoE), α2-microglobulin, and immunoglobulin G may also contribute to the pathophysiological processes leading to WMLs (Lammie, 2002; Nag, 2003). Another pathological process of deep small cerebral vessels particularly affecting the small veins of periventricular areas is known as venous collagenosis (Moody et al., 1995). This process, which has received limited attention compared to arteriolosclerosis in relation to the pathophysiology of SVD, is primarily associated with WMLs. Recent biological studies have demonstrated an association between alterations in RNA transcription in multiple genes involved in cell cycle, proteolysis, immunological modulation, and apoptosis and WMLs (Simpson et al., 2009). Genetic factors also appear to play a role in the development of WMLs, with reported heritability of up to 55–80% (Carmelli et al., 1998; Atwood et al., 2004).

The results of GWAS also provide powerful tools to identify genes related to complex multifactorial traits such as WMLs. In a recent meta-analysis of GWAS involving 9,361 individuals of European descent and belonging to seven community-based cohorts, six novel SNPs were identified on one locus of chromosome 17q25 (Fornage et al., 2011). These novel SNPs encompassed six known genes including the WW domain binding protein gene (WBP2), two tripartite motif-containing genes (TRIM65 and TRIM47), the mitochondrial ribosomal protein L38 gene (MRPL38), the Fas-binding factor 1 gene (FB1), and the acyl-coenzyme A oxidase 1 gene (ACOX1; Fornage et al., 2011). The genes are known for being involved in a broad range of biological processes including innate immunity, cell cycle regulation, vesicular trafficking, neuroprotection, and apoptosis (Vandeputte et al., 2001; Meroni and Diez-Roux, 2005; Ozato et al., 2008). This provides the first step toward characterization of biological mechanisms that influence the pathophysiology associated with WMLs (Fornage et al., 2011). The multiple pathophysiological processes involved in the genesis of WMLs underscore the complexity of this phenotype.

Though WMLs were historically considered incidental findings of doubtful clinical significance, recent epidemiological studies have demonstrated that WMLs are associated with cognitive decline (van Straaten et al., 2006; Frisoni et al., 2007; Pantoni, 2008; Wright et al., 2008). Moreover, the combination of WMLs in patients with deep brain infarcts is also associated with more cognitive decline (Miyao et al., 1992; van Swieten et al., 1996). WMLs are seen in at least 30% of patients with AD and in 60% of patients with dementia (Steingart et al., 1987). Some studies have found that a greater burden of WMLs is also associated with incontinence, gait dyspraxia, and incident falls (de Leeuw et al., 2001; Baezner et al., 2008; Srikanth et al., 2009). An appraisal of 16 studies confirmed the association between WMLs and cognitive decline in different patient settings: hospital-based to population-based (Pantoni et al., 2007). Finally, a large meta-analysis of 46 observational studies, demonstrated that WMLs are associated with greater risk of future stroke, dementia, and death (Debette and Markus, 2010). Effective therapies targeting the development of WMLs based on the understanding of the pathophysiology and plausible molecular targets are desperately needed.

### CEREBRAL MICROBLEEDS

This phenotype of SVD refers to small deep or superficial hemorrhages of 2–10 mm in diameter seen by MRI (Greenberg et al., 2009; Shoamanesh et al., 2011; Wardlaw et al., 2013). The T2^*^ gradient echo sequence, and the newer susceptibility-weighted imaging (SWI), provide sensitive methods for detecting microbleeds (Tanaka et al., 1999). These lesions correspond to small collections of hemosiderin-laden macrophages around small perforating vessels (Greenberg et al., 2009; Shoamanesh et al., 2011; Wardlaw et al., 2013). Two types of cerebral microbleeds have been described in the literature (Vernooij et al., 2008). A pattern of deep sub-cortical microbleeds associated with vascular risk factors, and a greater burden of WMLs and deep brain infarcts, and a lobar pattern associated with CAA and the ApoE4 genotype (Vernooij et al., 2008). The prevalence of cerebral microbleeds in the general population is about 5% but could be as high as 23–44% in those patients that have suffered ischemic strokes, and 52–83% in those that have suffered intracranial hemorrhage (ICH; Cordonnier et al., 2007). Indeed, cerebral microbleeds are a strong predictor of future spontaneous and symptomatic ICH (Lee et al., 2004). The implications of SVD and cerebral microbleeds on clinical management in acute ischemic stroke are very important. Intravenous recombinant tissue plasminogen activator (i.v. rt-PA) is an effective therapy in the acute setting of stroke (The National Institute of Neurological Disorders and Stroke rt-PA Stroke Study Group, 1995; Wardlaw et al., 2009; Lees et al., 2010). In addition to older age, hypertension, and hyperglycemia, the presence of SVD and microbleeds has been associated with a greater risk of ICH (Neumann-Haefelin et al., 2006; Palumbo et al., 2007; Charidimou et al., 2013). There is some data on the effect of SVD in candidates for the extended t-PA window (3–4.5 h). However, a history of prior stroke is considered to be a contraindication for thrombolysis in this time window, and as mentioned previously, patients with a prior stroke have a higher prevalence of cerebral microbleeds (Charidimou et al., 2013). Finally, the role of antiplatelet or anticoagulant therapy in patients with SVD and microbleeds deserves comment. The presence of SVD and older age are each associated with ICH risk during antiplatelet therapy, as reported by the Stroke Prevention in Reversible Ischemia Trial (SPIRIT; Gorter, 1999). In a systematic review, the burden of microbleeds in warfarin users with ICH compared to other groups shows that microbleeds increase the risk of warfarin-associated ICH (Lovelock et al., 2010). Therefore, in these patient population, conventional secondary prevention strategies using antiplatelet or anticoagulation therapy requires a thorough analysis of the risk benefit ratio.

The correlation of cerebral microbleeds and cognition is a matter of current research. A greater burden of cerebral microbleeds has been associated with cognitive impairment (Werring et al., 2004), and some studies in subjects with CADASIL have found a greater burden of cerebral microbleeds are associated with worse functional ability (Viswanathan et al., 2010). A recent MRI-based study confirmed that the presence of cerebral microbleeds was associated with global cognitive dysfunction (Yakushiji et al., 2008) in independent adults with no evidence of neurological dysfunction. The mechanisms underlying the pathological association between cerebral microbleeds cognitive dysfunction and overt VCI remain unclear (Charidimou and Werring, 2012).

## FUTURE DIRECTIONS AND ONGOING RESEARCH

Small vessel disease is an important cause of stroke and VCI. Single component clinical trials targeting classic risk factors for both VaD and AD are ongoing. The ongoing SPRINT Memory and cognition IN Decreased hypertension (SPRINT-MIND)^1^ study will attempt to determine if lower systolic blood pressure (SBP) goals influence the rate of incident dementia and MCI, global and domain-specific cognitive function, and small vessel ischemic disease. The Aspirin in Reducing Events in the Elderly (ASPREE) trial is evaluating the effect of daily aspirin on incident dementia and physical disability. The sub-study of the Secondary Prevention of Small Subcortical Strokes (SPS3) trial is looking at the rate of cognitive decline in patients treated with aspirin and/or clopidogrel (Benavente et al., 2011). The Efficacy and Safety Study of Nimodipine to Prevent Mild Cognitive Impairment After Acute Ischemic Strokes (NICE) is testing the hypothesis that the calcium channel blocker nimodipine may be associated with less cognitive decline and VaD. Additional ongoing trials are considering the effect of multi-component interventions. The Finnish Geriatric Intervention Study to Prevent Cognitive Impairment and Disability (FINGER) trial is using lifestyle counseling including nutritional guidance, increased physical activity, cognitive training, increased social activity, and intensive monitoring of vascular and metabolic risk factors to prevent VCI. The Austrian Polyintervention Study to Prevent Cognitive Decline After Ischemic Stroke (ASPIS) trial is using Intensive control and motivation for better compliance with medication, regular blood pressure measurements, diet changes, and physical activity versus standard stroke care to prevent cognitive decline. The Prevention of Dementia by Intensive Vascular Care (PREDIVA) trial uses intensive vascular care with visiting a practice nurse every 4 months to assess vascular risk factors, including hypertension, hypercholesterolemia, diabetes, overweight, smoking, and level of physical exercise; intervention: lifestyle and medical to prevent dementia and disability. Finally the Prevention of Decline in Cognition After Stroke Trial (PODCAST) is testing the hypothesis that intensive blood pressure (SBP < 125 mm Hg) and/or lipid-lowering [low density lipoprotein (LDL) < 2.0 mmol/L] versus moderate blood pressure (SBP < 140 mm Hg) and LDL (<3.0 mmol/L) is associated with less cognitive decline, AD, and/or VaD.

Novel molecular interventions using genetic approaches include targeting of proteins related to specific pathways of acute and chronic ischemia. Candidate genes include NOTCH3, HTRA1, and APOE ε4 (Dichgans and Zietemann, 2012). There is also interest in novel risk factors for dementia such as the role of free radical oxygen formation in mediating some of the deleterious effects of aging, hypertension and CAA-β-amyloid deposition on small vessels (Iadecola et al., 2009).

## CONCLUSION

The association between cerebral SVD and VCI has been deduced mostly from case series and observational studies. Pathophysiological studies of conditions affecting the small vessels of the brain and leading to SVD have suggested plausible molecular mechanisms involved in vascular damage and their impact on brain function. Similarly, MRI technology has helped us to better define surrogates of disease that may be used as markers of disease onset, progression, and impact of future therapies. GWAS studies have also elucidated some potential molecular mechanisms associated in pathophysiology of certain phenotypes of SVD. However, there is much that still needs to be clarified in understanding the pathophysiology of VCI in relation to SVD. No specific therapy currently exists for SVD-related stroke, and, more importantly, standard treatments for acute ischemic stroke are not associated with better outcomes in patients with SVD. Effective therapies to limit or halt the progression of SVD are needed. Until more is known in reference to the pathophysiological mechanisms of SVD and results of ongoing clinical trials become available, treatment of vascular risk factures such as hypertension should be the focus of prevention strategies. Based on the results of recent clinical trials antiplatelets or combination antiplatelets should be used with caution in this patient population.

Future prevention strategies will depend primarily on the refinement of our understanding of the pathophysiology of this condition. The results of clinical trials targeting known risk factors for VCI are forthcoming.

## Conflict of Interest Statement

Fred Rincon: None; Clinton B. Wright: royalties from UpToDate, Inc. for review articles on vascular dementia.

## Footnotes

1 http://www.sprinttrial.org
